# Feeding Behavior of Subadult Sixgill Sharks (*Hexanchus griseus*) at a Bait Station

**DOI:** 10.1371/journal.pone.0156730

**Published:** 2016-05-31

**Authors:** Bryan McNeil, Dayv Lowry, Shawn Larson, Denise Griffing

**Affiliations:** 1 Seattle Aquarium, Seattle, WA, United States of America; 2 Washington Department of Fish and Wildlife, Olympia, WA, United States of America; North Carolina State University, UNITED STATES

## Abstract

This is the first in-situ study of feeding behaviors exhibited by bluntnose sixgill sharks. Bait was placed beneath the Seattle Aquarium pier situated on the waterfront in Elliott Bay, Puget Sound, Washington at 20m of water depth. Cameras and lights were placed around the bait box to record sixgill shark presence and behavior while feeding. Analysis of feeding behavior revealed that sixgills utilize a bite comparable to many other elasmobranchs and aquatic vertebrates, have the ability to protrude their upper jaw, change their feeding behavior based on the situation, and employ sawing and lateral tearing during manipulation. The versatility of their feeding mechanism and the ability of sixgills to change their capture and food manipulation behaviors may have contributed to the species’ worldwide distribution and evolutionary success.

## Introduction

Sharks are found in every ocean of the world and are typically at the top of the food web of those systems. Many of the larger sharks are wide ranging, occurring in most of the world’s oceans such as the broadnose sevengill (*Notorynchus cepedianus*), the great white shark (*Carcharodon carcharias*), the blue shark (*Prionace glauca*), and the bluntnose sixgill shark (*Hexanchus griseus*) [1,2,3]. Despite the widespread distribution of large sharks, they are all at risk of population decline because of life histories that include late maturity and low reproductive capacity with the potential of overexploitation by humans, i.e. harvesting, finning, by-catch, and entanglement [2,4]. The population status of, and the impact of fisheries on, many sharks remains unknown, prompting their listing as either data deficient (broadnose sevengill shark), vulnerable (great white shark), or near threatened (blue and sixgill sharks) by the International Union for the Conservation of Nature (IUCN) [5]. Effective management of most shark species has proven difficult because little is known about their basic biology and ecology.

Despite the worldwide distribution of sixgill sharks, their biology and behavior are not well studied. This is possibly due to their deepwater habitat preference [6] and the lack of accessible, consistent, and concentrated abundance. Sixgills are found worldwide in temperate and tropical seas at the continental and insular shelves [2] to depths of 2490m [7]. These sharks have a diel vertical migration pattern, staying deeper in the daytime and coming shallower in the evenings [8]. Dunbrack and Zielinski [9] found that in the Strait of Georgia, British Columbia, sixgills were present at depths of 20-40m from June to September. They are thought to be generalist feeders preying and scavenging mainly upon teleosts, chondrichthyans, cephalopods and crustaceans [6,10]. As they grow larger, marine mammals become an increasingly important part of their diet[10].

Prey capture bites among elasmobranchs, and other aquatic vertebrates, are accomplished by one or more of the following methods: ram feeding, biting, and suction feeding [11]. Ram feeding is when a predator attacks its prey with an open mouth and over-swims the prey in order to trap it in its jaws or engulf it entirely [12]. Oral manipulation, i.e. biting, occurs when the predator approaches the prey with an open mouth, stops, and traps the prey within the jaws. Biting is then followed by sequential processing bites to facilitate ingestion by either swallowing the prey whole or reducing it into manageable pieces [13]. Suction feeding occurs when subambient pressure within the buccal cavity is generated, resulting in an inertial force that carries the prey, and an entrained parcel of water, towards the mouth for capture [11,14,15].

While elasmobranchs of the orders Squaliformes, Lamniformes, and Carcarhiniformes are mainly ram feeders, often with situation-specific suction and biting components particular to their prey [13,16–22], the Heterodontiformes, Orectolobiformes, Squatiniformes, and Batoidea are primarily suction feeders [23–27]. These groups of elasmobranchs are well represented by studies of prey capture, feeding ethology and/or feeding kinematics, while studies of feeding within the order Hexanchiformes are lacking.

Congruently, these studies have also focused on the elasmobranchs that possess evolutionarily derived jaw suspensoriums (orbitostyly, hyostyly, and euhyostyly) while studies of the *in situ* performance of an evolutionarily conserved elasmobranch jaw mechanism, i.e. amphistyly/orbitostyly, seen in Hexanchiformes [28], are minimal. It is suggested that the sixgill jaw suspension form is limited in extension (30° gape angle) and permit only mid-water/epibenthic feeding unless scavenging [29]. Modern shark jaw suspensoriums allow, to a varying degree, the upper jaw to protrude from the mouth during feeding. In upper jaw protrusion, the upper jaw disarticulates from the chondrocranium to increase the functional reach of the jaw apparatus, by decreasing predator-prey distance and jaw closure time [30–34]. Some studies suggest that sharks within the Order Hexanchiformes cannot protrude the upper jaw because it is limited by the presence of a postorbital articulation between the chondrocranium and palatoquadrate, as opposed to jaw suspensoriums of other sharks that lack a postorbital articulation [35–37]. While others suggest that disarticulation of the hexanchiform jaw may be limited [28,38] and have been seen in *Notorynchus cepedianus*, sevengill sharks[39]. It would seem that clarification in regards to the sixgill shark on this subject is needed.

When feeding, a fish may utilize various, often subtly distinct, sets of integrated motions (kinematic techniques) to capture different types of prey in diverse situations, which is known as modulation. Several shark species have the ability to modulate prey capture and feeding behavior. McNeil [2003, unpublished data] found that young of the year brownbanded bamboosharks (*Chiloscyllium punctatum*) modulated capture kinematics when consuming live prey versus dead prey by significantly decreasing the timing and duration of several motions, presumably as a consequence of the potential elusivity of the live prey. Lowry and Motta [26] found that the whitespotted bambooshark (*Chiloscyllium plagiosum*), exhibited variability in feeding kinematics in response to prey type/elusivity but would show only true behavioral modulation in overall approach velocity to elusive prey. While significant modulation seems to be present in sharks, there are still some inconsistencies on the subject. The leopard shark (*Triakis semifasciata*), has been found not to modulate feeding behaviors when attacking prey [21] using ram-suction captures, conversely Lowry *et al*. [22] found that they do modulate behavior to use a ram dominated attack on truly elusive prey. The ability to modulate prey capture techniques amongst Hexanchiformes is not known. However, there has been few documented descriptions of sixgill sharks feeding. They have been seen feeding in a head-down, tail-up position while feeding on benthic prey via submersible [29] and scuba [40]. While attempting to feed, the sixgills were seen positioned at a 45–60° angle above the substrate while feeding on jonah crabs (*Cancer borealis*) [29] and nearly vertical while also pinning lingcod (*Ophiodon elongatus*) down with the snout [40]. A sixgill has also been seen, via submersible, sitting on the bottom close to bait and sucking the bait into its mouth [41]. These observations suggest that the sixgill can employ multiple prey capture techniques and possibly modulate feeding behavior.

After prey capture a predator may need to manipulate the prey in order to facilitate ingestion, if the prey is too large to swallow or its orientation hinders entrance into the buccal cavity. In manipulation, prey is trapped between the predator’s jaws while employing a combination of head, fin, and tail movements, often accompanied by changes in body orientation. Generally, prey capture is shorter in duration then prey manipulation [16,20] due to the added time needed to shear prey into smaller pieces or orient it appropriately. How Hexanchiform sharks manipulate their prey for ingestion is unknown.

Sixgill sharks are occasionally common in the Puget Sound, especially in Elliot Bay near Seattle. There is currently very limited, anecdotal data on how sixgills feed and what modes of prey capture and manipulation or strategies are utilized during feeding. In Elliot Bay, this study sought to answer the following questions:

1. How do sixgills capture food (biting, ram, suction, or combinations of the previous)?
2. How do the kinematics of prey capture compare to other sharks?
3. What is the nature and extent of prey manipulation?
4. Is modulation in feeding behavior present?

## Methods

### Experimental procedure

The Seattle Aquarium is located on Pier 59/60 in Elliot Bay, giving it unusual access to typically deep-dwelling sixgill sharks. On the west end of Pier 59, an underwater research station, consisting of a diver cage built into the pier pilings, a lighted bait box, and an array of underwater cameras was constructed to observe these sharks as part of an ongoing education and scientific program. Other published studies have occurred utilizing this research site [42,43]. For this study, archived underwater video footage (VHS) from previous Seattle Aquarium shark research events was analyzed for sixgill feeding behavior. Usable footage had been previously catalogued from research events targeted at taking biopsies and tagging individual sixgills for genetics and population abundance studies[42,43]. Sharks were attracted to the site using bait, however the experimental setup was not specifically designed for a feeding study. The authors were aware of potential limitations of the footage, but it did produce repeatable, quality images of sixgill sharks feeding under controlled conditions, representing a unique opportunity to study feeding behavior. Footage was collected bimonthly from January 2003 to May 2005. Since sixgills were most abundant during the summer months [32], video footage chosen for analysis came from an event recorded from 1800–0600 on July 31- August 1 and August 1–2, 2003. This footage was chosen for its high quality, in part due to water clarity, and high number of shark sightings.

Sharks were attracted by bait set at a depth of 20m, in a box surrounded by four fixed lights (2-Super-SeaLite and 2-Multi-SeaLite; Deep Sea Power & Light, San Diego, CA), and five fixed cameras (3-Delta Vision Industrial and 2-Deep Blue Pro; Ocean Systems Inc., Everett, WA) for video documentation of shark behavior. Footage from one 3-Delta Vision Industrial camera, which generally provided a lateral view of the heads of feeding sharks, was chosen to derive all feeding behavior data, while the other cameras were used to confirm motion variables when sharks changed orientation during feeding. The footage was recorded at 29.97 frames per second (fps). The bait was placed at the bait station via SCUBA and consisted of carcasses of dogfish (*Squalus suckleyi*), salmon (*Oncorhynchus spp*.), and halibut (*Hippoglossus stenolepis*). Un-tethered bait was placed into a 0.8x0.8x0.5m bait box (benthic bait) while 20-L frozen, buoyant boluses (mid-water bait) were tethered to the bait box ~1m above it. The frozen mid-water bait was tethered with ~1m long steel cable to the bait box with a buoy inside the frozen bolus to maintain buoyancy. Fresh bait was placed at the beginning of each recording cycle, at approximately 1800hrs each day. As the event progressed, the mid-water bait would become smaller due to sharks consuming it and, as it thawed, pieces fell down into the bait box.

The sex of each shark was determined by the presence/absence of claspers using an upward facing camera. Length was determined by gross *in-situ* morphometrics. In parallel and planar lateral views with the bait box, the fifteen sharks in this study showed a head length (i.e., tip of the snout to the last gill slit) to total length ratio of 1:5. The head length to total length ratio was applied to determine total length for each animal using the bait box as a reference grid.

### Strike Composition Data Collection

Food acquisition strikes enacted by sixgill sharks on the bait produced data for two different bait types, benthic and mid-water baits. A strike was defined by a shark attempting to bite one of the bait types, and the activity following capture of the bait including manipulation bites/shearing and overall body movements and orientation. Conclusion of a strike occurred when the shark obtained a manageable portion of the bait and could freely swim away from the bait station. Manipulation of the bait during a strike was included in the data set while manipulation of fragmented portions of bait was not, because sharks would normally swim out of camera range. Ultimately, sixty strikes were recorded from fifteen individuals (thirteen females and two males) that ranged from 2–3.5m in length. Depending on viewing quality, obstruction by other bait items, other sharks, the bait box, etc., the number of samples per variable was sometimes less than the total number of strikes.

To determine if sixgill sharks exhibit different behaviors when consuming benthic vs. mid-water bait, strikes were compared between the two target types. Behavior was quantified for both bait types by the following:

- Food capture methods (Ram, suction, and/or biting).
- Number of bites per strike.
- Total time of strike in seconds (Once the cranium was lifted during approach to the bait).
- Manipulation techniques utilized (Shaking, shearing, ripping, etc.).
- Total time of bait manipulation in seconds (Time spent trying to remove a manageable chunk of bait, time reorienting the bait with other bites excluded).

For food capture method determination, gross forward progress of the shark towards the bait and the movement of the bait were used. Ram-suction Index (RSI) values (i.e., ratio of ram vs. suction feeding, based on lateral movement of the predator vs. movement of the prey item) [12] could not be determined due to the severely limited capacity to control the approach behavior of sharks and obtain a suitable orthogonal view. Ram feeding was present if the shark made forward progress with no obvious suction affecting the movement of the bait, i.e., the bait did not move. However, if there was no forward progress by the shark, and the bait moved into the jaws of the shark, suction feeding was indicated. If both the shark and bait moved toward one another, ram and suction components were deemed present. Lastly, to acquire the bait via biting, a shark must have stopped swimming and ended all forward progress close to the bait and not cause any visible suction while grabbing the bait with its jaws. Once the bait was seized, the sharks would manipulate the bait via subsequent bites and shearing, which was also described.

### Bite Kinematics Data Collection

To qualify for detailed kinematic analysis, footage of a bite needed to meet these quality criteria: shark and bait must be illuminated, adequately visible, feeding mechanism must be in a resting state, unobstructed, and orthogonally oriented to the camera. This meant that only bites on the mid-water bait qualified as the approach angle for benthic bait was sub optimal. Ten bites total during the event met the criteria for kinematic analysis, and these bites came from six subadult sharks (four females and two males) ranging from 2–3.5m in length. Only prey capture and recapture bites were kinematically quantified. Footage was analyzed using Redlake Motion Imaging Software 2.30.0. Kinematic displacement variables were not measured in each frame of a feeding sequence but were rather measured for several frames immediately preceding and following visually discerned maximum displacements. Maximum gape was quantified on seven bites by measuring from the center of the upper and lower jaws during peak displacement; angle of the jaws during maximum gape were measured.

Kinematic variables quantified included: (1) onset of cranium elevation and (2) mandible (Meckel’s cartilage) depression, both with an onset of 0ms; (3) cranium elevation duration; (4) time of complete cranium depression; (5) duration of mandible depression; (6) time of complete mandible elevation; (7) labial cartilage/fold extension onset, (8) maximum extension, and (9) duration of extension; (10) time of complete labial cartilage retraction; (11) onset of upper jaw (palatoquadrate) motion, (12) protrusion, (13) protruded duration, and (14) time of complete retraction; (15) time bait was seized, (16) in the mouth; (17) and maximum gape (defined as the time when both the cranium and mandible were at their peak displacement); and (18) total bite time, which occurred when the food was seized and both cranium and mandible returned to their resting state.

Due to the high prevalence of densely aggregated plankton in the Puget Sound, quality of VHS recordings, and severely limited capacity to control the approach behavior of sharks in the open experimental environment, not every kinematic variable could be quantified for each of the bites selected for review. Sometimes loose cranial skin folds obstructed the view of the labial cartilages or upper jaw as they shifted around the chondrocranium during a strike. Values for those variables that could be quantified were combined across all ten bites to create a composite bite profile of a sixgill bite with descriptions of variability, thus interindividual variation is not addressed in this study.

## Results

### Strike Composition

Of the 60 strikes analyzed, 58 occurred during 1800-2300hrs (33 on July 31, 25 on Aug 1), with two strikes from 2300–0600 July 31-Aug 1, and none from 2300–0600 Aug 1–2. For comparison, the sharks struck the mid-water baits 40 times, while there was 20 strikes on the benthic bait. The sharks seemed to arrive at the bait station in pairs, however, the largest (3.5m) and the smallest (2m) individuals each arrived alone.

It was quite common for sharks to lower their pectoral fins right before a strike and keep them down during manipulation. This action seemed to stop forward progression to allow for easier foraging and in some instances was used to pull the shark backwards for repositioning or to avoid collision with the bait box. A few times the pectoral fins lifted the body of the shark up off the bait box. Commonly, the sharks rolled their eyes posteriorly, concealing the lens throughout a strike. The sharks would only roll their eyes back when objects were close to the eye or when something contacted the body abruptly (e.g., a biopsy or tagging dart). It was also common to have the sharks swim pass in and out of camera view as well as bumping into the bait or bait box with their snout then pass out of view of the camera. This bumping may have been investigative behaviors and/or just accidental collisions.

Both bait types were dominantly captured by ram feeding (Table 1); however, benthic bait had higher incidences of both suction and biting. When near the bait box or the sea floor, there were obvious substrate-influenced suction effects during the capture bite [21,33]. When the sharks were positioned properly for adequate viewing, upper jaw protrusion was visible in 24 strikes (not present = 3, unknown = 33), in either capture and/or manipulation bites.

**Table 1:** Observed behavioral composition of strikes on benthic and mid-water bait by sixgill sharks.

		Capture Methods^a^				Strike and Manipulation Data^b^				Shearing Methods^c^			
Bait Type	Ram	Suction	Bite	Ram and Suction	n	Total Strike Time in seconds	Time of Manipulation during Strikes in seconds	Number of bites per strike	Twist	Tear	Twist and Tear	None	n
**Benthic bait**	45.5% (5)	27.3% (3)	18.2% (2)	9.1% (1)	11	9.7 ± 7.9 (14)	0.7 ± 0.5 (2)	3.6 ± 3.1 (16)	11.8% (2)	*NA*	*NA*	88.2% (15)	17
**Mid-water bait**	86.7% (26)	3.3% (1)	6.7% (2)	3.3% (1)	30	23.7 ± 13.7 (38)	8.9 ± 5.8 (21)	7.7 ± 4.3 (39)	47.4% (18)	15.8% (6)	26.3% (10)	36.8% (13)	38

^a^ Percentage of capture methods used on both bait types. Captures not in view of the camera or of low quality are excluded. Number of each capture method observed is shown in parentheses.

^b^ Means of strike and manipulation duration, and number of bites per strike, with standard deviation according to bait type. Sample size of variable denoted in parentheses.

^c^ Percentage of shearing methods used during manipulation per strike on both bait types. Shearing not in view of the camera, or of low quality, is excluded. No observations indicated by *NA*. Number of observations of each method observed is shown in parentheses.

Prior to the first bite of a strike, the sharks would frequently exhibit slow, moderate cranial and mandible activity before reaching the bait, as an incomplete bite or a preparatory bite. The cranium and mandible would open slowly and not reach max extension and may not return back to the resting state prior to the capture bite. The snout would frequently come into contact with the bait, and the upper or lower jaw would only sometimes contact the bait. Sometimes only the cranium would lift with no mandible movement. Upper jaw protrusion was not seen during these preparatory bites. Of the strikes for which presence of a preparatory bite could be evaluated, it was present in 79% of strikes on mid-water baits and 50% on benthic baits.

Once the bait was seized, sharks would manipulate the bait by biting for repositioning, or by shearing off pieces. During manipulation, the sharks would not always remain horizontal, sometimes adopting a head-down posture and taking a position vertically above the bait.

Strike durations were considerably shorter on the benthic bait ($\overset{-}{x}$ = 9.7s, *SD* = 7.9s) than the mid-water bait ($\overset{-}{x}$ = 23.7s, *SD* = 13.7s) (Table 1), however both exhibited variability. The benthic bait also elicited fewer bites per strike ($\overset{-}{x}$ = 3.6) than the mid-water bait ($\overset{-}{x}$ = 7.7). Commonly during strikes on mid-water baits, the bait would rotate around the tethering point, causing the sharks to abandon the bait briefly and then recapture it. The recapture bite differed from the repositioning bites by returning the feeding mechanism to its resting state prior to recapture, essentially making it identical to the initial capture bite. Rate of successful strikes resulting in manageable bait acquisition was higher for the benthic bait (94%) than the mid-water (72%).

**Fig 1:**
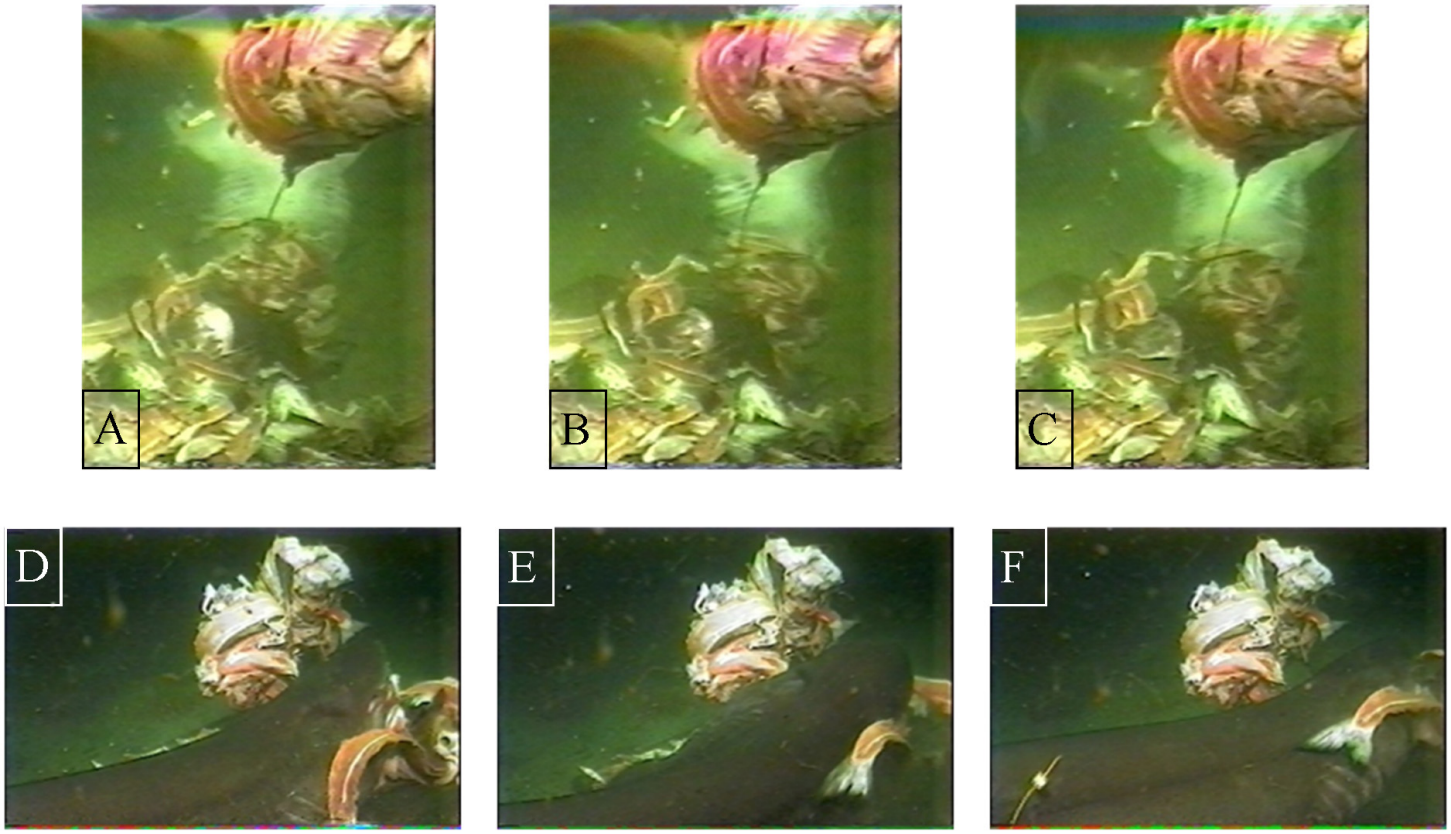
Two sixgill sharks manipulating bait by twisting. A-C (2.9m male) twisting while in the head-down vertical orientation with ventral surface facing the camera. The abdomen is partially obscured by the mid-water bait. A-center of swing, B-rotation right, C-rotation left past center. D-F (3.3m female) twisting during horizontal orientation with the head of the shark to the right. D-center of swing, E-rotation left, F-rotation right past center, visual tag seen on the shark in lower left hand corner.

**Fig 2:**
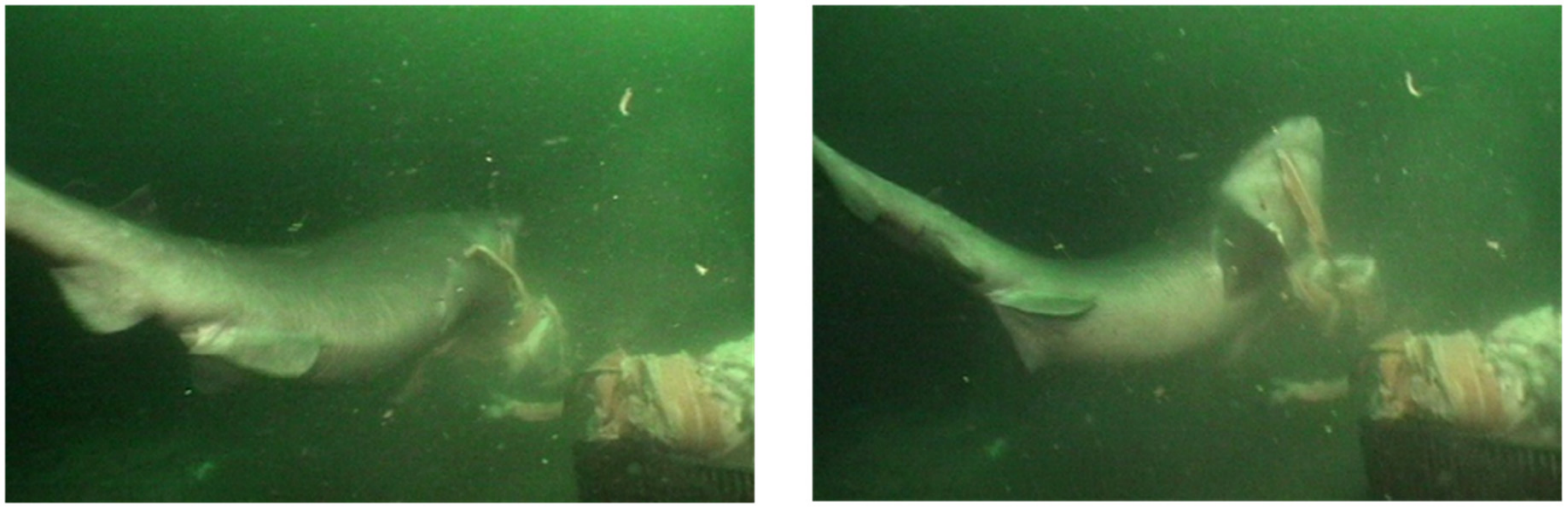
Sixgill shark employing unilateral tear to remove a salmon carcass from mid-water bait. 3.3m female.

During shearing manipulation, sixgill sharks employed two techniques: twisting and unilateral tearing. Twisting was the most commonly used shearing technique on the mid-water bait, while any shearing was rarely required for consumption of the benthic bait (Table 1). Mean shearing durations on the mid-water bait averaged longer ($\overset{-}{x}$ = 8.9s, *SD* = 5.8s) than the shearing required on the benthic bait ($\overset{-}{x}$ = 0.7s, *SD* = 0.5s); in fact, only two instances of shearing on the benthic bait were seen (Table 1). Twisting was performed in short arcs, centered at the midline of the upper jaw (Fig 1). Twisting seems to start behind the head, while the rest of the body may or may not twist along with the head. During each arc the lower jaw would swing about ≤45° in either direction. The teeth along the upper jaw sink into the bait and act as an anchor while the lower jaw cuts away at the bait, producing a manageable chunk of food. A unilateral tear was strictly used at the last moments of the strike on mid-water bait, commonly excising a bolus of food and completing the strike. Unilateral tearing was performed when the mouth was almost closed with the morsel held within the buccal cavity. The tear was performed by rapidly swinging the trunk dorsolaterally once, almost always removing the chunk of food from the larger bait item (Fig 2). Of the 16 tearing events, the sharks employed more than one unilateral tear only 3 times (19%) to complete a strike.

### Bite Kinematics

**Fig 3:**
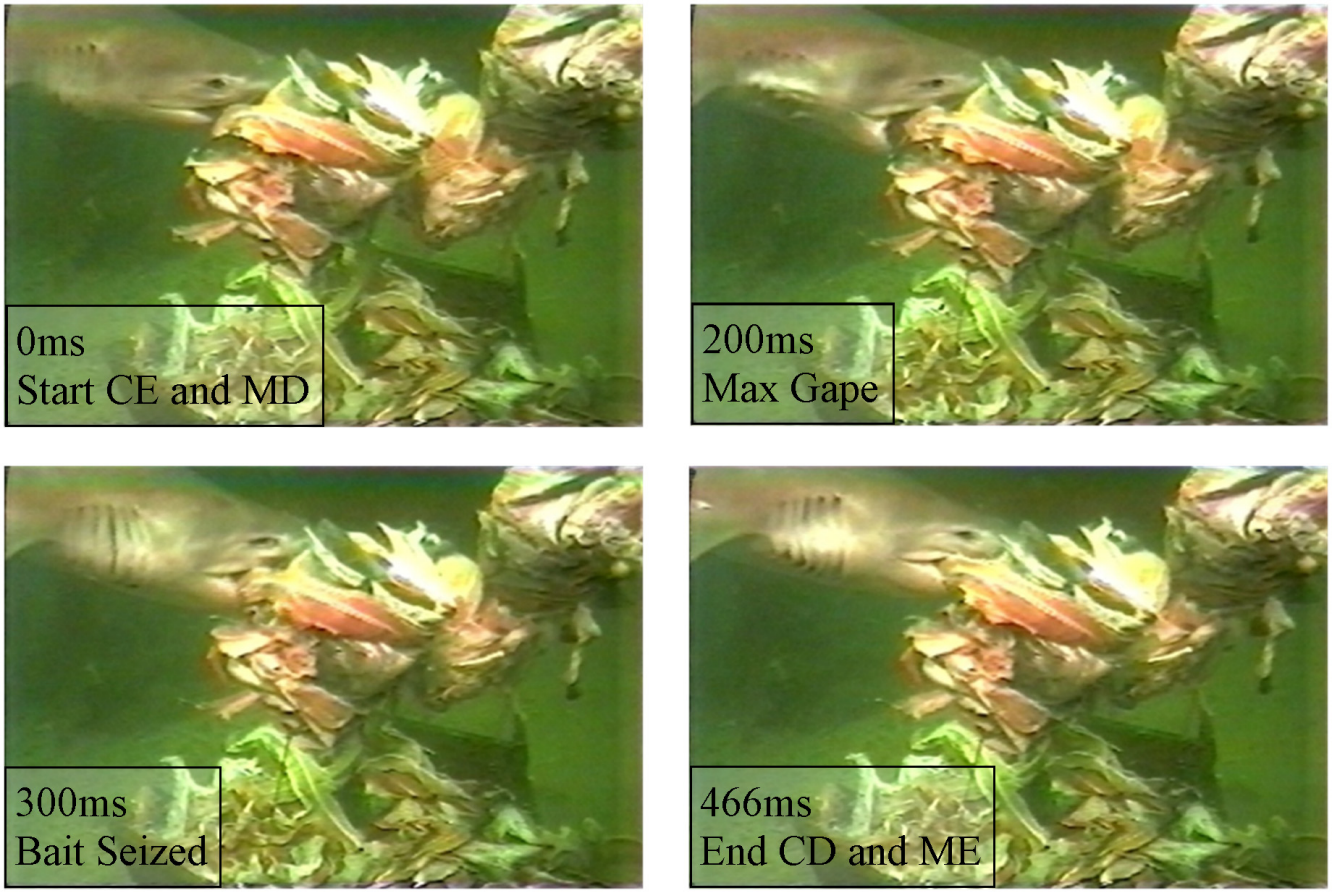
Bite sequence of a 2.9m male sixgill. CE = start of cranial elevation, MD = start of mandible depression, CD = end of cranial depression, ME = end of mandible elevation. Upper jaw protrusion was obscured from view.

**Fig 4:**
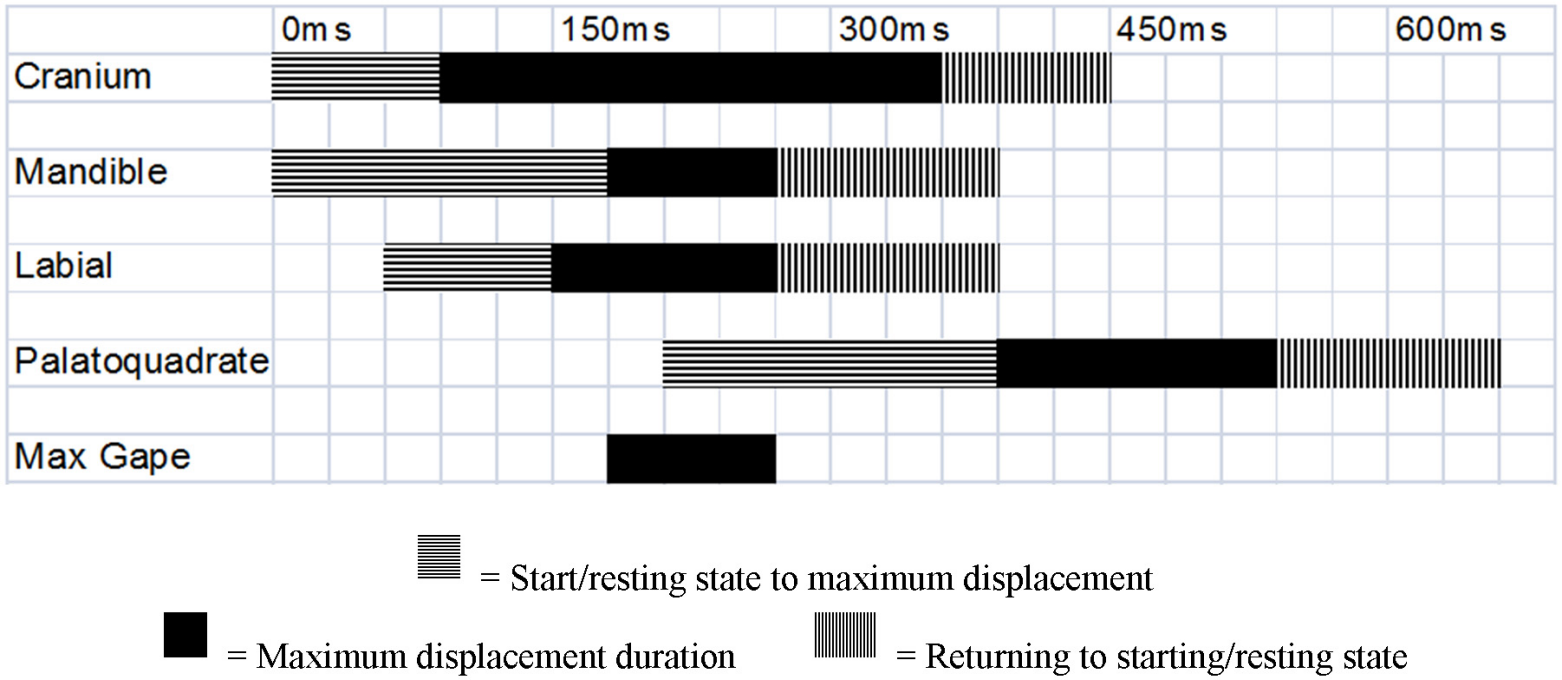
Composite diagram of kinematic variables from a single representative bite. Bite from a 3.3m female sixgill shark.

Of all strikes recorded, bites from six individuals fulfilled the requirements for detailed kinematic analysis, resulting in ten usable bait consumption events. All bites were on mid-water bait; eight of the bites were recapture bites, while two were initial capture bites. All bites started with cranial elevation and mandible depression, which were initiated simultaneously in all cases (Table 2, Figs 3 and 4).

**Table 2:** Bite kinematics.

Group	Factor	Mean Durations (ms) ± SD	Time elapsed during bite (ms) ± SD	n
Cranium	Elevation	163 ± 65	163	8
(Onset 0ms)	Max Elevation Duration	174 ± 123	319	9
	Depression	204 ± 72	534	8
Mandible	Depression	220 ± 72	220	10
(Onset 0ms)	Max Depression Duration	47 ± 23	267	10
	Elevation	220 ± 118	487	10
Labials	Onset	96 ± 60	96	8
	Extension	108 ± 58	204	8
	Max Extension Duration	229 ± 118	338	8
	Retraction	359 ± 190	492	8
Upper jaw	Onset	225 ± 57	225	4
	Protrusion	183 ± 123	409	4
	Max Protrusion Duration	117 ± 118	551	2
	Retraction	489 ± 171	701	3
Bait position	In mouth		145 ± 31	3
	Seized		371 ± 111	9
	Maximum Gape		215 ± 67	9
	Total Bite Time		547 ± 160	10

Kinematic variable means combined from six sharks from ten bites captured via ram. Activity of each kinematic group was observed in all bites analyzed; however number of observations of each factor is included because some events were out of view of the camera or of low quality. Durations and events are given with ± Standard Deviation to show variability.

During the expansion phase, the cranium would rise until 163ms while the mandible would depress to 220ms. The bait would enter the mouth before the mandible and cranium would reach their peak (145ms). The labial cartilages would extend as a consequence of the mandible and cranium activity, with an onset of 96ms, and would reach maximum extension at 204ms. Mean maximum gape occurred at 215ms. Measure of maximum gape averaged 37% of head length (n = 7, range 31–42%) and opened at an average angle of 50° (n = 7, range 45–69°). The upper jaw, if in view, would protrude from the cranium frequently, however it was only in view for a few bites included in the kinematic analysis. At 225ms, after the cranium and mandible were at their peak displacement, the upper jaw would protrude from the cranium anteroventrally, initiating the compressive phase.

**Fig 5:**
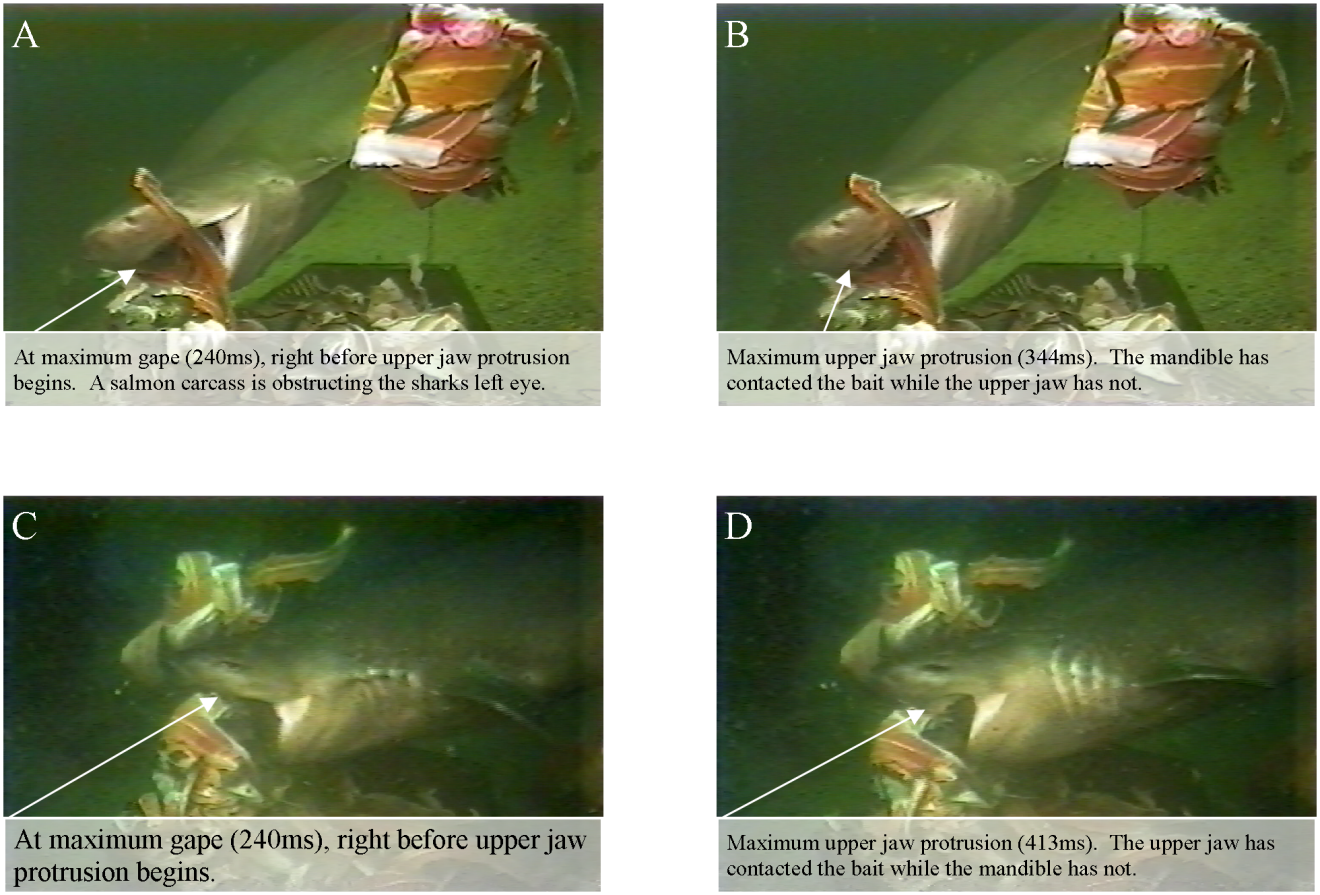
Examples of upper jaw protrusion and labial extension. Images of a 3m female (A-B) and a 3.3m female (C-D), respectively.

The mandible and cranium would then start closing at 267ms and 319ms, respectively, while the labial folds would stay at their peak displacement until 338ms. The bait was seized by 371ms, as the upper jaw protruded anteroventrally. The upper jaw reached maximum protrusion at 409ms (Fig 5). Upper jaw protrusion was measured as approximately 10% of the total maximum gape (relative measure of gape at time of max gape vs. relative measure of maximum upper jaw protrusion). The mandible would then be fully elevated by 487ms, with the labials fully retracted at 492ms. The recovery phase would start once the cranium was fully depressed at 534ms; however, the upper jaw would still be at its peak displacement into the recovery phase until 551ms.

Total bite time was determined once the mandible, cranium, and labials had completed their cycles (547ms). The upper jaw would be fully retracted at 701ms, ending the recovery phase.

## Discussion

### Food Capture

Sixgill sharks employed all three capture methods when feeding on the two bait types in this study, demonstrating versatility of the feeding apparatus, and modified their behavior according to bait type (Table 1). While the sixgills changed their behavior and capture method on each bait type it was difficult to determine why the sharks changed their behavior. The two bait types were drastically different from each other in terms of spatial orientation, size, and whether or not they were tethered. These variables confound each other, making it difficult to determine why the sharks changed their behavior. These bait types were used because this research site was already in use for another study. However, it is clear that sixgill sharks do change their feeding behavior based on the situation. The authors provide potential explanations for changes shark behavior below.

Ram capture was the most often utilized method on both bait types. Capture of the benthic bait, however, had higher incidences of the use of suction and biting behavior. Ram capture seemed most appropriate when approaching a prey item if the forward path after/during prey capture is not obstructed by an object or the sea floor. This was the case for mid-water bait approaches and exit paths, while benthic bait was sitting either in the bait box or on the sea floor, which created paths that may have been obstructed by the bait box itself, diver cage, other sharks, or the sea floor.

While elements of suction could be seen in a number of bites, suction as the primary means of feeding was only seen on benthic bait. Production of that suction was most likely aided by substrate effects on the bait [21,33]. Sixgills can produce a large gape, allowing for the potential for a large parcel of water to be enveloped within the buccal cavity (Fig 2) and passed posteriorly through the gill slits. This allows for ingestion of large food items and likely helped produce suction effects seen during this study. However, it is still unknown if sixgills can produce enough suction alone to have a significant effect on elusive prey or prey that is grappled to a substrate. Sixgills that have been seen attempting to feed on jonah crabs [29] utilized suction to capture them, however the suction created was not enough to lift the crab into their jaws. In this study, when suction alone was used on dead benthic bait, its usefulness was obvious, but the extent of how much was aided by substrate effects or even size effects of bait size is unknown.

Interestingly, there was twice the amount of strikes on mid-water bait than benthic bait. This may be due to the accessibility and visibility of the mid-water bait versus the benthic bait which was visually and physically obstructed by the bait box. By contrast, the mid-water bait had to be manipulated much more than that of the benthic bait that yielded 94% successful strikes on the benthic bait versus 72% of mid-water strikes. However, sharks that had already fed and returned to the site to feed again were still more likely to strike the mid-water bait. Perhaps accessibility and visibility of a prey item is more important than the amount of manipulation needed to feed. It should be noted that the mid-water bait was a frozen bolus of bait that thawed over time, while the benthic bait was already thawed when placed at the sea floor. There may be an olfactory difference between the two bait types that might have influenced the sixgills’ behavior.

### Manipulation

Strike and shearing duration, number of bites, and amount of manipulation during a strike were much higher for the mid-water bait than the benthic bait. This was due to the mid-water bait being large, partially frozen, and tethered, while the benthic bait was untethered and generally consisted of smaller, individual pieces of bait. In fact, the size of the mid-water bait was larger than most of the sharks maximum gape, unless previous pieces were partially excised or thawed away from the center of the bolus. Those factors lengthened feeding duration due to complexity associated with handling the mid-water bait. However, by having the mid-water bait tethered, the sharks revealed their array of prey manipulation techniques. While twisting (Fig 1), the lower jaw swings side to side while pivoting along the upper jaw that had pierced into the bait, while the rest of the body either swung slightly or seemed to maintain balance and stability, i.e. pectoral fin drop and/or movement/rigidity of the tail. The upper jaw was used as the center of rotation allowing the lower jaw to saw into the bait and excise a manageable chunk of bait. The heterognathus dentition of sixgills allows for this; the lower teeth are compressed, short but wide, and serrated while the upper teeth are compressed, long but thin with small cusps. Whitenack and Motta [44] found that the lower teeth of sixgill sharks work well as cutting teeth, and in this study that notion is supported. As long as the upper teeth pierced the bait, this sawing motion was quite effective.

Sixgills feed mainly on teleosts and chondrichthyans but are also generalist predators feeding on cephalopods, crustaceans, and with marine mammals becoming an increasing part of their diet as they grow larger [6,10]. Most likely, this type of manipulation is key when biting through large prey items including blubber of pinnipeds and whale carrion. The sawing motion then allowed the lower jaw to progress into the partially frozen boluses well enough to remove large chunks from the bait. As the lower jaw would swing back and forth along the prey item, the arcs of each swing seemed to be ≤45° and never exceeded 90° in either direction. This twisting method has also been seen in the Greenland shark (*Somniosus microcephalus*) [George Benz, personal communication] when excising a bolus from a large food item, such as whale carrion. Greenland sharks have similar heterodonty, saw-like teeth in the lower jaw and thin, piercing teeth in the upper jaw. Dissection of the sixgill sharks feeding apparatus has demonstrated a broad articulating facet on the dorsomedial aspect of the upper jaw that braces against the ventral aspect of the postorbital cartilage [D. Lowry, unpublished data]. This bracing of the upper jaw along the postorbital cartilage may support the upper jaw during twisting manipulation, helping it maintain its anchor point while the lower jaw pivots back and forth.

Sixgills sometimes employed a unilateral tear during manipulation, alone or along with twisting. The lateral tearing of the bait was very uniform and always concluding the strike, resulting in eventual consumption of the bait (Fig 4). No vigorous head shakes, as seen in other elasmobranchs [13,16,19] were observed. A tear was performed only when the mouth was almost fully closed, with the manipulated piece of bait within the buccal cavity. This technique might only be used when the shark assumes that there is no need to manipulate the prey anymore. If more than one tear was attempted, the following tears would only occur once the whole body rotated after the force of the preceding lateral tear had concluded. This type of manipulation, along with waiting for the body to follow-through after a tear, may allow for maximum force to be applied to the bait while conserving energy. This does not discount, however, that sixgills could employ lateral shakes of the head to manipulate prey. That behavior may be within the sixgills ability but was not seen during this study.

As a note, these sharks were also seen to use a “spit-suck” manipulation on smaller, loose bait items for reorientation, winnowing, and transport. This type of manipulation has been seen in suction feedings nurse sharks, *Ginglymostoma cirratum* [24,45] but was typically on food that was larger than their gape. Most of the "spit-suck" events in this study occurred in suboptimal areas on the videos and could not be kinematically analyzed. However, this was quite frequent and it seems to utilize slower movements of at least the cranium and mandible. In a few instances, the sharks had engulfed sunflower sea stars (*Pycnopodia helianthoides*), that were preying on loose bait items. The sharks would use the spit-suck technique with the sea stars mostly within the buccal cavity. The sharks were able to transport the bait items to the pharynx and expel the sea stars.

### Bite Composition

Due to the large size and typically sluggish nature of sixgill sharks, individuals were expected to exhibit slow approaches, slower bites, and more prolonged manipulation events than that observed in other elasmobranchs as a consequence of predator size on feeding performance [21,46]. Total bite time and manipulation confirmed those expectations and allowed for sufficient analysis at 29.97fps. The total bite time was slow compared to smaller elasmobranchs but seems to be as fast as that of the great white shark, (Table 3). Size effects cannot completely explain the bite speed of sixgills because the sharks studied were all subadults ranging from 2–3.5m, as compared to the animals filmed by Tricas [18], which were 3–3.5m great whites. As of note, the potential for muscle performance to be slower at colder temperatures (water temperature at time of study was 11–12°C) could also contribute to the slower bite time than other sharks studied which have been in tropical or temperate waters [13,16–27,39]. Overall, sixgills reside in colder, most often deeper waters and has a conserved jaw apparatus, but are still capable of executing a comparatively rapid bite. The coordinated, quick nature of these movements is likely vital for the capture of live prey and it is unlikely that the sixgills observed in this study were performing at the limit of their feeding bite speed capacity due to the use of nonelusive bait.

**Table 3:** Comparisons of bite kinematic variables and manipulation between sixgill sharks and sharks from Orders Lamniformes, Squaliformes, Carcharhiniformes, and Orectolobiformes respectively.

	Sixgill, Hexanchus griseus	Great White, *Carcharodon carcharias* ^a^	Dogfish, *Squalus acanthias*^b^	Lemon, *Negaprion brevirostris*^c^	Nurse, *Ginglymostoma cirratum*^d^
Prey Capture Method	Ram/Suck (Bite also, but no kinematic data)	Bite and Ram	Ram/Suck	Ram	Suction
Cranial Activity	Simultaneous with lower jaw	Simultaneous with lower jaw	After lower jaw begins to depress	After lower jaw begins to depress	Not frequent
Upper Jaw Initiates	After lower jaw is depressed	After lower jaw is depressed	After lower jaw begins to elevate	After lower jaw begins to elevate	Not visible during study
Upper jaw fully protruded	Before lower jaw is elevated	Before lower jaw is elevated	Before lower jaw is elevated	After lower jaw is elevated	Not visible during study
Part of feeding apparatus that concludes activity first	Lower jaw	Lower jaw	Lower jaw	Cranium	Lower jaw
Total Bite Time (ms)	701; 547^e^	985; 443^e^	302	309	100
Manipulation observed	Twisting and lateral tear	Lateral head shake	Lateral head shake	Lateral head shake	Lateral head shake

^a^—[17, 18],

^b^—[16],

^c^—[13],

^d^—[24],

^e^-Mean bite duration not including the cranium and upper jaw reaching resting position.

The sixgill sharks in this study also had an average maximum gape angle of 50°, up to 69°. This information suggests that the jaw apparatus of sixgill sharks is not as restricted as to the 30° gape angle previously reported [29]. Movement of the mandible and labial folds during a bite resembles a typical bite observed in other elasmobranchs [16,24,46,47,48]. However, sixgills do differ in timing and movement of the cranium as well as the timing of upper jaw protrusion in most other sharks (Table 3). Bites were always initiated by elevating the cranium simultaneous with the depression of the mandible, while other elasmobranchs either intermittently elevate the cranium or do not elevate it at all [13,22]. These timing and use parameters actually resemble the bite of great whites, where they initiate a bite with a snout lift for each capture bite [18]. Timing of upper jaw movement activity also resembles that found in great white sharks [18] and sandtiger sharks (*Carcharias taurus*) [49], in that full extension of the upper jaw occurs before the mandible is elevated. This is not the case for several species of shark [16,24], which tend to contact food items with both their upper and lower jaws nearly simultaneously.

While sixgills were repositioning the bait via consecutive bites during manipulation, activity of the cranium, mandible and upper jaw were modulated. During rapid bites (bites that reached max displacment before 215ms, max gape), sometimes the cranium would remain elevated, with the upper jaw still protruded, relying on the mandible activity to reposition the bait. This modulation of the timing and decoupling of these kinematic variables has also been seen in other sharks [13, 17].

### Preparatory bite

Preparatory bites are characterized by limited and slow movements of only the cranium and mandible, which may or may not have returned to their resting state prior to the capture bite. This behavior is similar to the normal under water pass reported in the great white shark, where the sharks would open their mouths partially about 1m away from the bait [17]. Once the snout touched the food, the great whites would depress their lower jaw to engulf the bait; both cranial and mandible displacement were present but not as pronounced as a normal bite and did not include upper jaw protrusion. It was suggested that the great white was waiting for a tactile sensory input from the snout before initiating a feeding action as the bait was not visible once the sharks were close [17]. With the sixgill, however, the snout or the jaws did not necessarily need to come into contact with the bait before performing a capture bite.

Since food was seized with a capture bite following the preparatory bite, the preparatory bite may aid the shark’s approximation of how to approach/seize a prey item. In this study, the sharks were feeding on differing bait items based on size and spatial orientation, that may not represent natural feeding conditions and may have been prudent of the sharks to approximate their bite and approach. In nature, this behavior could give potential prey valuable time to escape, as with other investigative behaviors [50]. Assuming sixgills have the ability to modulate their bite mechanics, this technique may only be present in the capture of slow or dead prey as well as prey that may not be aware of the shark’s presence (i.e. stealth or dark water).

This type of bite is similar to investigatory bites seen in great white sharks [50,51]. Both investigatory and preparatory bites seem to be investigative behaviors, investigatory bites have been suggested to aid the great white shark in ascertaining palatability [51]. This is not the case with a preparatory bite in the sixgills. While in preparatory bites, the sixgills performed incomplete bites where the shark may or may not make contact with the bait, and frequently preceded a capture bite. This is not to suggest that sixgill sharks do not perform investigatory bites, rather to say that these types of bites are different and performed for different reasons.

### Upper jaw protrusion

In upper jaw protrusion, the upper jaw disarticulates anteroventrally from the chondrocranium to increase the functional reach of the jaw apparatus, by decreasing predator-prey distance and jaw closure time [30,31, 32,34]. It has been hypothesized that hexanchiform sharks are considerably restricted in mobility/protrusability of the jaw apparatus based on the degree of connectedness between the upper jaw and the chondrocranium, directly via an articular facet on the dorsomedial aspect at the center of the upper jaw and via the ethmopalatine ligament, as well as indirectly via the hyoid arch [35–37]. However, it has also been thought that disarticulation of the hexanchiform jaw may be possible [28,38] and has been seen in sevengill sharks [39]. The sixgill sharks in this study did protrude the upper jaw quite frequently. Of the kinematic bites measured, protrusion of the upper jaw accounted for 10% of the max gape. This study confirms that sixgills do possess the ability to protrude their jaws as do seven gill sharks [39]. The protrusion of the upper jaw in sixgills does seem to disarticulate from the cranium proportionately ventrally more than it does anteriorly. Protrusion of the upper jaw was also independent of cranial and labial activity as seen in the kinematic timings (Table 2) and for its lack of presence in preparatory bites. The upper jaw would start to protrude once maximum gape was achieved, upper jaw protrusion would peak right before the mandible would be fully elevated.

While the cranium and mandible close, mouth closure time was decreased by this protrusion, it is not as effective as seen in other shark species [13,16,39] who can protrude the upper jaw farther. Upper jaw protrusion in sixgills may still be restricted due to the degree of connectedness to the chrondocranium as previously proposed [35–37]. Despite this, performance of this protrusion is most likely important for sixgills in capturing more elusive prey than dead prey or frozen bait as offered in this study.

### Modulation

Variability among shark feeding kinematics is common [16,20–24,26,30,46,47], and the sixgill shark exhibits variability as well. In fact, all kinematic variables were quite high and there are multiple reasons why. The maximum gape angles had a notable range which the cranium and mandible did not displace the same amount proportionately each time, therefore either extending or shortening all timing sequences. It has also been shown that kinematics will vary over ontogeny in leopard and whitespotted bamboo sharks [22,26,46,47]. The sixgills in this study ranged from 2–3.5m in length, most likely representing different age ranges. It could not be determined if there were shark size (age) effects in this study due to the low sample sizes of kinematic data. The two bait types, mainly the mid-water bait, did have confounding variables (tethered, size, orientation, etc) that could add to variability due to altered approach, position, and performance.

Despite all of the above potential reasons for variability, the sixgills did show versatility of the feeding mechanism which most likely added to the variability. The preparatory bites performed by the sixgills showed independent use of the cranium and the mandible, sometimes displacing one or both. During manipulation, the sixgills repositioned the bait with consecutive bites sometimes elevating the cranium with the upper jaw still protruded and utilizing the mandible alone to reposition the bait. This modulation of the timing and decoupling of these kinematic variables has also been seen in lemon sharks (*Negaprion brevirostris*) and great white sharks [13,17]. This gives the impression that modulation could occur within the jaw apparatus of the sixgill shark. Historically, there has been challenges to reporting modulation in shark feeding kinematics [20,23], however modulation has been seen during prey capture [13,17,22] and processing [23,45]. The aforementioned range of gape angles could also be due to modulation, potentially the sixgills only opened their mouth as wide as needed and not in a stereotypical fashion, like that seen in suction feeding sharks [24,45]. But even the nurse shark (*Ginglymostoma cirratum*) has shown modulation during manipulation [24,45] using a spit-suck technique. A similar technique was utilized by the sixgill sharks during manipulation, using slow movements of at least the cranium and mandible to expel sunflower sea stars that had grappled onto the bait for which the sharks were trying to eat.

The authors suggest that the sixgill shark has the ability to modulate its feeding behavior, even though the sample size for kinematic analysis is small. The repeated presence of decoupling of multiple feeding kinematic variables gives evidence that sixgills are capable of modulation.

### Research Site

The research setup employed here was utilized for other studies on sixgill sharks, and as a result the video archives were not ideal for a typical feeding study. While the authors are aware of these limitations, enough data have been extracted to generate a summary of generalized feeding behaviors and techniques used by sixgill sharks, which was previously described only by opportunistic anecdotal accounts. In fact, because the setup was not typical, some feeding behaviors might not have been observed under traditional experimental setups. However, with some alterations, more comprehensive studies could be conducted at this site.

### Implications and Ecological Impact

This study has shown that sixgills have the ability to modulate their feeding behavior, can significantly protrude the upper jaw, and share kinematic profiles to more derived sharks like great whites. This supports the notion that the sixgill shark’s amphistylic/orbitostylic jaw suspension, does not perform much different than that from more derived jaw suspension types.

The ability to utilize ram, suction, and biting capture methods and protrude the upper jaw suggests that sixgills have the ability to feed on diverse, and elusive prey. This complements the known prey items of sixgills [6, 10,52], but information on what these subadults are feeding on in the Puget Sound is still lacking. However, stable isotope analysis of carbon and nitrogen on subadult sixgill shark tissue in Puget Sound suggest that these animals feed primarily on mobile benthic invertebrates, such as Dungeness crab (*Metacarcinus magister*) [Greg Williams, personal communication]. It is thought that sixgills alter their diet as they grow [6,10] but there is no data on how this presumed dietary switch affects their feeding and/or foraging behavior.

This study supports that sixgill sharks modify their food capture behavior depending on target type and orientation (benthic vs. mid-water); and that they have the ability to modulate bite kinematics depending on conditional circumstances.

## Supporting Information

S1 Data Set Data set. Minimal data set associated with this article.
(XLSX)

S1 Video Two sixgill sharks feeding at the bait station. This video shows the research site with the cage for diver protection to the right with the bait station typically center; stationary cameras for video analysis can be seen multiple times. This video includes two sixgills feeding at the bait station, both with visual tags. A biopsy is performed on the first shark that swims by the camera for another study. Almost all the behaviors discussed in this article are on display here. Video taken by SCUBA diver.
(MP4)

S2 Video Representative strike 1. A feeding event with a 2.9m male sixgill approaching the bait station from the right. The shark starts the strike in an horizontal plane, parallel with the sea floor and goes into a head-down, tail-up position during manipulation, almost upside down at one point. View of the shark is obstructed by mid-water bait multiple times throughout the video. The sixgill in this video exhibits preparatory bite, capture bite, manipulation bites, and shearing manipulation via twisting and tearing. Footage from the raw video was used for analysis.
(MP4)

S3 Video Representative strike 2. A feeding event with a 3.3m female (with a visual tag) approaching from the right. This female does not exhibit a preparatory bite but does show a prey capture bite, rolling of the eyes, manipulation bites, and shearing manipulation via twisting and tearing. Upper jaw protrusion is seen during manipulation. Footage from the raw video was used for analysis.
(MP4)

S4 Video Representative strike 3. A 3m female approaches the bait station from below and starts a strike on mid-water bait once it swims over the bait box. The strike includes a preparatory bite, capture bite, eye rolling, upper jaw protrusion and manipulation/transport bites. The sixgill then ingests the bait and swings around and strikes the benthic bait. View is obstructed during this strike mostly due to position but manipulation bites and transport bites are seen. Footage from the raw video was used for analysis.
(MP4)

S5 Video Sixgill manipulating a sea star. A male sixgill shark (with a visual tag) manipulating a sunflower sea star while trying to eat the bait the sea star was holding. Video taken by SCUBA diver.
(MP4)
